# Data Needs and Uses for Older Adult Health Surveillance: Perspectives From State Health Agencies

**Published:** 2005-06-15

**Authors:** Christopher Maylahn, Sam Alongi, Jeanne Alongi, Margaret J Moore, Lynda A Anderson

**Affiliations:** Division of Chronic Disease Prevention and Adult Health, New York State Department of Health; Third Sector Strategies, Sacramento, Calif; Association of State and Territorial Chronic Disease Program Directors, Sacramento, Calif; Centers for Disease Control and Prevention, Atlanta, Ga; Centers for Disease Control and Prevention, Atlanta, Ga, Rollins School of Public Health, Emory University, Atlanta, Ga

## Introduction

As the population of older adults in the United States increases, state public health agencies are confronted with new opportunities and challenges (1). These agencies recognize the need to form new partnerships and develop effective and innovative approaches for addressing the needs of older adults (2). Public health initiatives to promote healthy aging and reduce the burden of chronic conditions are currently underway and include partnership efforts by public health organizations and aging services agencies to implement evidence-based health promotion programs, disseminate information about aging issues, and strengthen their ability to expand these efforts (3). To ensure that informed decisions are being made and to measure progress toward health goals related to older adults, state health agencies interpret and disseminate data on the health of older adults (4). These agencies rely on *surveillance* — the continuous, systematic collection and analysis of health data — a core tool of public health practice (5). However, previous work to develop indicators that measure whether a community promotes health and well-being among older adults (6) and to create a picture of older adult health has not focused on public health issues (7) or included state-level data (8). Similarly, the development of indicators for chronic diseases (9,10) has not focused on older adults. To fully understand the breadth and depth of older adult health from a public health perspective, these efforts must be combined to produce a core set of public-health–related indicators to measure older adult health at the state level.

As the first step toward our ultimate goal of creating a core set of indicators, the Centers for Disease Control and Prevention (CDC) and the Association of State and Territorial Chronic Disease Program Directors (CDD), a national public health association of the chronic disease program directors in each state, began to explore indicators that are particularly relevant to healthy-aging activities within state health agencies. The CDD established the Aging Surveillance Advisory Committee to assess state public health agencies' needs, preferences, and priorities for data to monitor older adult health and develop recommendations for potential core indicators.

In this article, we describe the process for exploring the state perspective on surveillance of older adult health, the results of our research, the way in which the findings helped shape the indicators selected for *The State of Aging and Health in America 2004* report (11), and potential next steps for creating a core set of indicators.

## Exploring the State Perspective

The CDD Aging Surveillance Advisory Committee convened an expert panel via teleconference in January 2003. Panel members were selected on the basis of their affiliation with federal, state, and local public health agencies; academic institutions; clinical care; national public health organizations, aging services organizations, or both; and philanthropic organizations, as well as their knowledge of surveillance, epidemiology, quality-of-life issues, aging issues, and public health programming. The panel was asked to focus on four broad areas: 1) appropriate age groups for older adult health surveillance, 2) criteria for including indicators, 3) essential subject areas for aging surveillance, and 4) audience needs and composition. The minutes of the conference call meeting were subsequently shared with panel members to ensure accuracy and solicit additional feedback.

We used the information from the expert panel to develop a survey of state health agencies. All 50 states and 8 territories (collectively referred to hereafter as *states*) were invited to participate. The survey asked for information about the following: 1) the characteristics or indicators of older adult health that currently are being monitored in the state, 2) the ways in which data are being reported and used (with examples of reports, if available), and 3) the indicators of older adult health that most need to be assessed. If a state did not submit a completed survey, up to three follow-up e-mails were sent.

Concurrently, we scanned the relevant literature to identify data sources and methodological approaches, current uses of surveillance data, and other aspects of surveillance that may have affected the ability of state health agencies to collect and use information about older adult health. The scan included published literature, conference abstracts, key informant resources, and reports from state health agencies and other organizations.

## Results and Recommendations

### Recommendations of the expert panel

The expert panel comprised 22 individuals that represented a broad range of expertise in the field of healthy aging. The panel recommended beginning surveillance of older adults at 50 years of age so that it would include the transition from employment to retirement and continue beyond the traditional boundary of 85 years. Three themes emerged in the panel's discussion of criteria for choosing indicators. First, panel members preferred using indicators that were linked to highly prevalent conditions such as arthritis, osteoporosis, and Alzheimer's disease. Second, they agreed that potential indicators should be sensitive enough to be able to monitor the effects of public health interventions (e.g., changes in the number of healthy days). Finally, they recommended that the criteria for selecting indicators should address the concerns of a diverse group of stakeholders (e.g., practitioners, researchers).

The expert panel recommended several potentially useful subject areas for the indicators, including traditional measures of morbidity and mortality, use of services, and the economic impact of health conditions. They agreed that indicators were necessary for the general areas of functional status, disability, mental health, and social connectedness. More specific subject areas that were deemed important were cognitive function, dementia, depression, abuse, hospice use, hospitalizations, and chronic diseases.

The panel concluded that disseminating the data to practitioners was critical for surveillance results to be useful in developing and evaluating aging-related activities. The recommended audiences for the data are broad and include staff members of state-level chronic disease programs and state units on aging, policy makers, local service agency representatives, and the general public.

### Survey of state health agencies

Twenty-one (36%) of the state public health agencies returned completed surveys. The population, geography, and degree of involvement in chronic disease and aging issues varied among the responding states.

Most often, the states relied on Behavioral Risk Factor Surveillance System (BRFSS) data (100%), mortality statistics (95%), and hospital discharge data (86%) for aging surveillance, although these data sources were not always sufficient to provide estimates for substate regions. Nine of the 21 states (43%) reported that they collected and reported data on issues such as depression, abuse, and institutionalization for long-term care among older adults. Others reported collecting data on cancer prevalence and mortality, women's health, caregiving, and the characteristics of those who seek health care for arthritis and osteoporosis.

Four respondents shared reports on the health of older adults. The content of these reports varied from basic statistical summaries to special reports on key issues of concern. For example, Maine's report, *Assessing and Monitoring the Health Status of Maine's Elder Women: The Elder Women's Health Indicators Project* (12), provides data on the health of older women in Maine that can be used for targeting interventions, measuring progress, and informing decision makers.

Eight states (38%) identified indicators pertaining to quality of life and functional status as the most important for routine monitoring, and six states (29%) prioritized access to care and chronic conditions and impairments. The survey also included questions about barriers to monitoring the health of older adults. The most frequent responses included lack of resources (33%), fragmentation of systems (29%), and lack of personnel (24%).

### Literature scan

The literature scan confirmed that no public-health–oriented lists of healthy-aging indicators exist. A copy of the results of the literature scan is available on request.

## Application of Findings

The expert panel and survey findings resulted in the following four domains for organizing healthy-aging indicators that are relevant to public health:

- *Risk factors:* no leisure-time physical activity, overweight or obesity, low intake of fruits and vegetables, tobacco use, and lack of clinical preventive services
- *Contextual factors:* social connectedness and access to care
- *Quality of life:* self-reported quality of life and self-reported health status
- *Health outcomes:* life expectancy, mortality, prevalence of diseases and conditions, and hip fractures

These findings were presented in May 2003 at the Making Health Count for Older Americans summit, which was convened by the CDC and the Center for the Advancement of Health. This summit brought together stakeholders representing public health, aging services agencies, academia, health care, the news media, and the government. The discussion centered on currently available reports on the health of older adults (7,8), critical health indicators for older adults at the national and state levels, the feasibility of regularly producing a report on older adult health, and appropriate formats for such a report. Subsequently, the CDC, the Merck Institute of Aging & Health, and The Gerontological Society of America collaborated to compare the recommendations from the CDD and the summit with available data and the nation's Healthy People goals (13,14).

The results of the collaboration were synthesized into *The State of Aging and Health in America 2004* report, which includes an analysis of 15 key indicators related to older adult health for the 50 states and the District of Columbia (Table) (11). Using data from the BRFSS, the report provides information about the number of states that met the *Healthy People 2000* (13) targets and includes current data for each indicator; state data (rank and grade) are listed alphabetically. The report also includes examples of state activities, including Maine's report on older women's health. As a result, *The State of Aging and Health in America 2004* is more than a compilation of data — it is a reference tool and blueprint for public health and aging services professionals to use as they address the health needs of older adults in their own communities. *The State of Aging and Health in America* *2004* has been well received by its intended audience. This positive response is a testament to the need for such information.

In addition to being shared at the national summit, our findings were distributed to stakeholders in a report (15) and presented at the 17^th^ National Conference on Chronic Disease Prevention and Control in 2003. The findings are an important step in creating a core set of indicators related to older adult health. Overall, the results of the expert panel and state survey revealed that states were interested in a broad range of indicators on health and aging. Although many indicators are available in current data systems, some measures (such as measures of social connectedness and hip fractures) are not yet available at the national and state levels.

Several factors should be considered when reviewing our findings. First, the survey data are not generalizable. Furthermore, as with any survey method, the information obtained from state public health agencies may have been biased because of the collection method used, the type of follow-up performed, and the timing of the request.

## Next Steps

We have not yet reached our goal of developing a set of core indicators, and our findings highlight the need for more explicit criteria for selecting them. They also suggest that the best approach for meeting the needs of state health agencies and their partners may be to develop a menu of indicators within the various domains identified in this pilot work. Future work should also involve a larger, more diverse set of stakeholders to identify such a menu. In addition, future work may benefit from a conceptual framework such as the ecological model of health (16).

Our experiences during this process have shown us that state public health agencies and other stakeholders believe that surveillance on the health of older adults is a critical public health issue. We hope that the findings and issues raised in this article will stimulate additional work and move this important agenda forward.

## Figures and Tables

**Table:** Fifteen Key Indicators^a^ Related to Older Adult Health and Analyzed in The State of Aging and Health in America 2004^b^

• Physically unhealthy days
• Frequent mental distress
• Oral health: complete tooth loss
• Disability
• No leisure-time physical activity in past month
• Eating five or more fruits and vegetables daily
• Obesity
• Currently smoking
• Flu vaccine in past month
• Ever had pneumonia vaccine
• Mammogram within past 2 years
• Ever had sigmoidoscopy or colonoscopy
• Up-to-date on select preventive services
• Cholesterol checked within past 5 years
• Hip fracture hospitalizations

a Indicators derived from the recommendations of the Association of State and Territorial Chronic Disease Program Directors. Source: Aging Surveillance Advisory Committee, Science and Epidemiology Committee, Association of State and Territorial Chronic Disease Program Directors (15).

b Source: Merck Institute of Aging & Health and Centers for Disease Control and Prevention (11).
